# Deep learning domain adaptation to understand physico-chemical processes from fluorescence spectroscopy small datasets and application to the oxidation of olive oil

**DOI:** 10.1038/s41598-024-73054-y

**Published:** 2024-09-27

**Authors:** Umberto Michelucci, Francesca Venturini

**Affiliations:** 1 Research and Development, TOELT LLC, 8600 Dübendorf, Switzerland; 2 Computer Science Department, Lucerne University of Applied Sciences and Arts, 6343 Rotkreuz, Switzerland; 3 Institute of Applied Mathematics and Physics, School of Engineering, Zurich University of Applied Sciences, 8401 Winterthur, Switzerland

**Keywords:** Fluorescence spectroscopy, Excitation emission matrices, Deep learning, Transfer learning, Domain adaptation, Fine tuning, Olive oil, Food quality, Image processing, Biophotonics, Nutrition

## Abstract

Fluorescence spectroscopy is a fundamental tool in life sciences and chemistry, with applications in environmental monitoring, food quality control, and biomedical diagnostics. However, analysis of spectroscopic data with deep learning, in particular of fluorescence excitation-emission matrices (EEMs), presents significant challenges due to the typically small and sparse datasets available. Furthermore, the analysis of EEMs is difficult due to their high dimensionality and overlapping spectral features. This study proposes a new approach that exploits domain adaptation with pretrained vision models, along with a novel interpretability algorithm to address these challenges. Thanks to specialised feature engineering of the neural networks described in this work, we are now able to provide deeper insights into the physico-chemical processes underlying the data. The proposed approach is demonstrated through the analysis of the oxidation process in extra virgin olive oil (EVOO), showing its effectiveness in predicting quality indicators and identifying the spectral bands and thus the molecules involved in the process. This work describes a significantly innovative approach to deep learning for spectroscopy, transforming it from a black box into a tool for understanding complex biological and chemical processes.

Fluorescence spectroscopy is a central analysis tool in life sciences and chemistry, with applications ranging from environmental monitoring, food quality control, to biomedical diagnostics. Furthermore, it is employed at all scales, from single molecules to tissues and organs, from protein dynamics to in-vivo imaging due to its sensitivity and specificity^1–3^. Fluorescence excitation-emission matrices (EEMs), in particular, provide detailed insights into the absorption and emission characteristics of substances, thereby acting as an effective fingerprinting tool^4,5^. However, analysis of EEM data presents significant challenges because of its high dimensionality and the frequent presence of overlapping spectral features. These challenges are traditionally addressed with multivariate chemometric methods such as principal component analysis (PCA) and parallel factor analysis (PARAFAC)^6,7^.

Deep learning (DL) has shown the ability to enhance scientific insight through advanced pattern recognition across multiple disciplines^8^. However, the effectiveness of hidden layers, critical for feature extraction and pattern decoding, is highly dependent on having access to extensive and varied datasets^9^. Thus, despite its potential, DL is still not widely used to explain and interpret biological, physical, and chemical processes from spectroscopic data because of (i) methodological and (ii) data-related challenges. Methodologically, DL architectures are designed primarily for computer vision tasks, focus on model decision making rather than understanding the underlying processes, and do not account for the correlation between signals at different wavelengths, treating them as independent features^10–13^. Data-related challenges arise because spectroscopy datasets are often small or sparse, exhibit either high similarity among images or excessive variability, and are frequently unbalanced^14,15^. These challenges hinder the training of large neural networks (NNs), resulting in high variance and inadequate performance, thus undermining confidence in the model and its feature engineering capabilities. Additionally, the instability of NNs prevents effective linking of feature extraction in hidden layers to the phenomena being modelled, as the networks may overfit to noise rather than learning meaningful patterns from the data.

Here we describe a novel method for spectroscopy applications that works for small and sparse datasets and that transforms DL into a tool to understand biological or physico-chemical processes. The method we propose is based on domain adaptation and a novel interpretability approach. This is demonstrated by analysing the natural oxidation process that occurs in extra virgin olive oil (EVOO) during storage, which deteriorates its quality^16^ and reduces its beneficial impact against the risk of cardiovascular and all-cause mortality^17^. The quality of EVOO is assessed through a series of parameters defined by the United Nations Food and Agriculture Organisation and the European Union^18–20^. Among these, the quality indicators $K_{232}$ and $K_{268}$, corresponding to UV absorbance at 232 and 268 nm, respectively, were chosen for this study because they measure primary and secondary oxidation products.

This work presents two major contributions. The first contribution is the proposed domain adaptation approach to design and train a DL model that performs a regression task with exceptional performance even on a limited dataset. The approach is applied to the prediction of the $K_{232}$ and $K_{268}$ quality indicators from fluorescence EEMs. The second is the method to extract the internal representation learnt from the trained DL model without any a priori knowledge or feature engineering. This approach identifies the most relevant spectral bands, revealing the chemical components of EVOO that are involved in the oxidation process.

## Material and methods

**Fig. 1:**
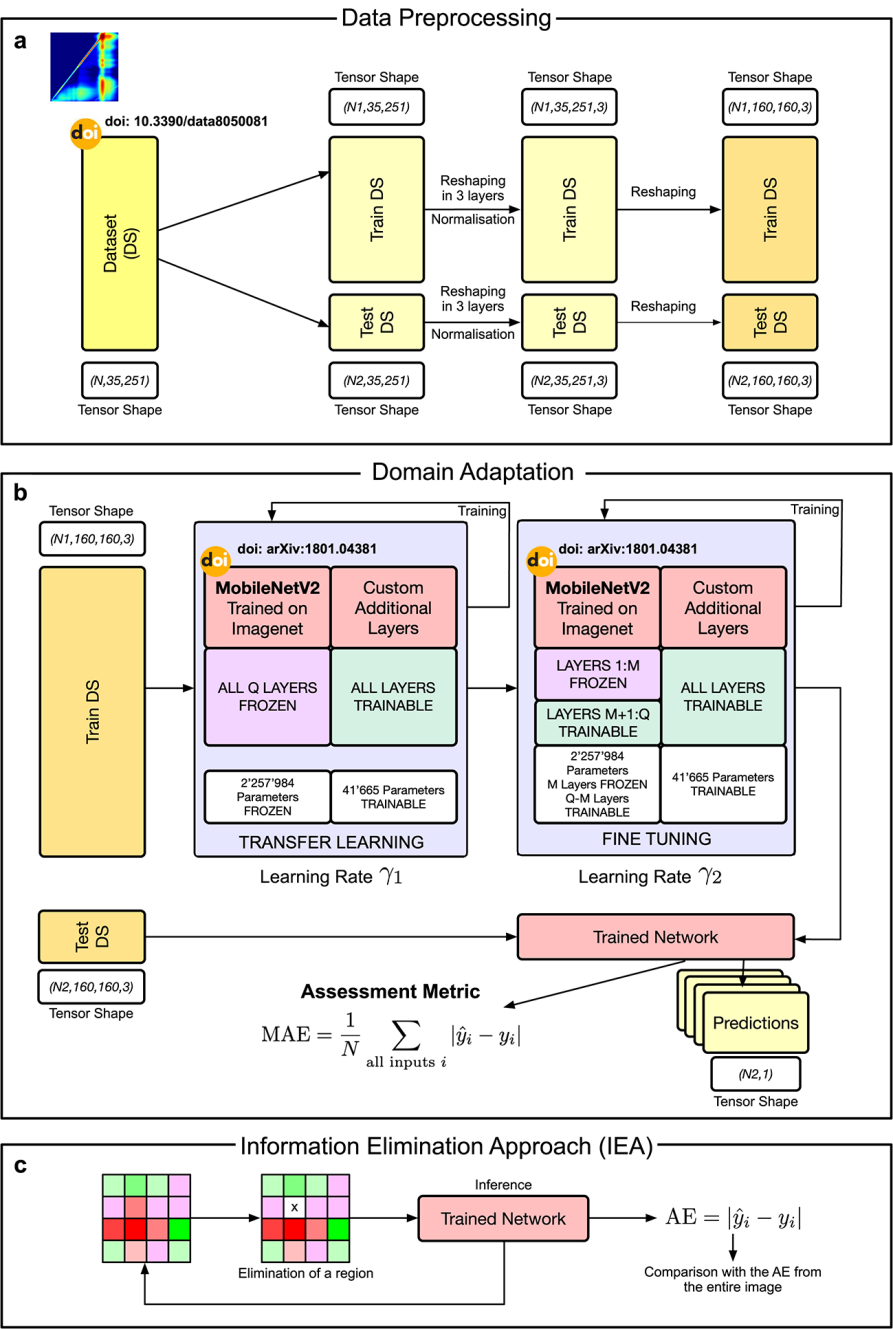
Overview of the phases of the machine learning approach. (**a**) The data preprocessing phase consists of splitting the dataset for the LOO approach, normalisation of pixel values, and preparation for the MobileNetv2 network input layer by reshaping and creating the three necessary layers. (**b**) The domain adaptation phase consists of transfer-learning and fine-tuning of the network using the training dataset. The trained network is then evaluated on the test dataset and its performance assessed through the Mean Absolute Error (MAE). (**c**) Information Elimination Approach (IEA) process diagram. doi references indicate the papers that describe some of the used components.

The proposed approach is divided into three steps, shown schematically in Fig. 1: data preprocessing, domain adaptation, and the information extraction approach. Domain adaptation (a technique where a model trained on data from one domain is adapted to perform well in another domain) improves the feature engineering capacity of neural networks^21^, but is, to the best knowledge of the authors, never been implemented in spectroscopy. The challenge of a small and sparse dataset is adressed by employing domain adaptation based on the network MobileNetv2^22^ (154 layers and ca. 3.5 million parameters) that has been pretrained on the ImageNet dataset^23^ (containing more than 14 million images and 20000 classes) and is known for its feature extraction capabilities. Domain adaptation is composed of two phases: transfer learning and fine tuning (Fig. 1b). This last step addresses the deficiency that the model has never seen any EEMs in its training data. Finally, to investigate the oxidation process, we introduce the Information Elimination Approach (IEA) (Fig. 1c). The IEA is the key to extracting information from the experimental observations. The method consists in removing part of the spectral information fed to the network as input and evaluating the model performance drop, thus assessing the importance of the removed information by its impact on the predictions.

### Fluorescence excitation-emission matrices and UV absorption dataset

The dataset comprises fluorescence spectroscopy EEMs and UV absorption spectroscopy data for 24 commercial EVOO, fresh, and in 9 oxidation stages. The oils were chosen to be as heterogeneous as possible in both the origin of production and the price to identify general features of the oxidation process. The characteristics of the oils are described in^24^. The oils underwent accelerated oxidation at 60 °C to investigate the impact of long-term storage. The effects of thermal degradation were evaluated at ten intervals over a time of 53 days. Fluorescence emission and UV absorption measurements at each stage provided insights into the oxidation process, which is responsible for changes in the oil’s chemical properties. The raw data for the fluorescence spectroscopy EEMs, acquired with an Agilent Cary Eclipse Fluorescence Spectrometer, are stored in CSV files, detailing fluorescence intensities across various excitation and emission wavelengths. Each file contains the fluorescence emission from 300 nm to 800 nm in 2 nm increments (251 values), under excitation at 35 wavelengths ranging from 300 nm to 640 nm in 10 nm increments. All data were acquired under identical conditions and at a controlled temperature of 22 °C within a thermalised sample holder, so the intensities are directly comparable. For UV spectroscopy measurements, the olive oil samples were diluted in isooctane and prepared according to EU regulations^18,19^. The analysis was carried out in sealed quartz cuvettes under identical conditions with an Agilent Cary 300 UV-Vis spectrophotometer and at a controlled temperature of 22 °C within a thermalised sample holder, collecting the extinction coefficients at four specific wavelengths: 232 nm, 264 nm, 268 nm and 272 nm.

The complete dataset is available for download and is described in detail in^24^ where the link to download the data can be found.

### Domain adaptation

Domain adaptation, in the context of machine learning, is a technique that involves adapting a model trained on data from one domain to perform well on data from another domain. This kind of approach is widely used for improving the model’s generalisation ability and performance on new, unseen data that differ from the model’s training data. To apply this approach in the case described in this work, it is necessary to prepare the data according to a set of steps. Firstly, the dataset was split for leave-one-out (LOO) cross-validation to ensure that each sample was validated exactly once. EEM’s pixel values (fluorescence intensities) ranged from 0 to 1000 counts, therefore, the intensity values were normalised in the dataset by dividing pixel values by a fixed value of 1000 to ensure no data leakage. Furthermore, data were reshaped to meet the input requirements of the MobileNetv2 network^22^, involving adjustments to $160 \times 160$ pixels, reformatting to three channels and conversion to an unsigned 8-bit integer format. The three channels were synthetically generated by triplicating the measured fluorescence intensity (so all three channels are identical), as depicted in Fig. 1a. Intensities from Rayleigh scattering, where the emission wavelength matches the excitation wavelength, were retained in the raw data. These intensities are typically considered not relevant, but we wanted to check if the network ignored them or not (the network correctly ignored the Rayleigh scattering). We also tested the results by removing the Rayleigh scattering (either by setting pixel values to 0 around it or by interpolating spectra around it) and we observed no difference in the results. These preprocessing steps are the key to adequately preparing the data for effective feature extraction and subsequent analysis using the pretrained MobileNetv2 network.

The model training is then performed according to the following phases, depicted in Fig. 1b.

1. Phase I - Transfer learning: in a first phase a network is built and trained according to the following recipe:
  Start with the MobileNetv2^22^ network with the weights obtained by training it with the ImageNet dataset.
  Remove all dense layers from the MobileNetv2 after the backbone.
  Freeze the backbone of MobileNetv2. The backbone will not be trained during this phase.
  Add the following sequence of layers to the MobileNetv2 backbone: (i) global averaging, (ii) a dropout layer with a factor of 0.2, (iii) a dense layer with 32 neurons with a ReLU activation function, (iv) a dense layer with 16 neurons with a ReLU activation function, (v) a dense layer with 8 neurons with a ReLU activation function, (vi) a dense layer with 1 neuron with an identity activation function.
  Train the network with the Adam optimiser. For all parameters $K_{232}$ and $K_{268}$ we used the following parameters: learning rate $\gamma =10^{-4}$, mean squared error (MSE) as loss function, mini-batch size $b=230$, and 1000 epochs.
2. Phase II - Fine-tuning: in a second phase the training proceeds according to the following steps.
  Unfreeze the last 54 layers of the MobileNetv2 backbone. During the training process, the initial 100 layers of the network remain frozen, with subsequent layers actively trained. The decision on the number of layers to unfreeze was based on multiple tests, which indicated minimal variation in performance when the number of frozen layers ranged from 100 to 120.
  Train the network with the Adam optimiser. For the parameters $K_{232}$ we used the following parameters: learning rate $\gamma =10^{-6}$, mean squared error (MSE) as loss function, mini-batch size $b=230$, for 500 epochs. For the parameters $K_{268}$ we used the following parameters: learning rate $\gamma =10^{-5}$, MSE as loss function, mini-batch size $b=32$, and 500 epochs.

The dataset comprises 24 oils and thus has a small size. To perform cross-validation, we chose the leave-one-out (LOO) approach. LOO cross-validation works by using all observations from the original sample except one (23 oils at their oxidation stages) as training data and the left-out single observation (one oil at its 10 oxidation stages) as validation data. This process is repeated so that each oil in the sample is used once as validation data. This method is particularly useful for our small dataset because it maximally uses the data for training while still ensuring that each data point is validated. The dataset is partitioned on the basis of individual oils to prevent data leakage, ensuring that all oxidation stages for a specific oil are grouped together in the same split.

### Information elimination algorithm

The Information Elimination Algorithm (IEA), presented in this paper, takes advantage of the representation of internal features of large NNs to explain and understand natural processes. It is crucial to emphasise that our aim is not to comprehend the thought process of the model, but rather to utilise the acquired knowledge to gain insights into the oxidation process. This algorithm systematically removes specific regions from the EEMs and observes the impact on the model’s performance, particularly focussing on the prediction errors. Specifically, the algorithm works as follows. Consider the trained model that has been validated on oil *j*. We proceed by first removing a region of $5\times 5$ pixels from the EEM of oil $j$ and assessing the impact on the prediction of $K_{232}$ and $K_{268}$ by recording the absolute error (AE). The process begins at the top left corner and proceeds horizontally towards the right edge. Upon reaching the end of a row, it continues from the leftmost side of the row immediately below, repeating this pattern until the entire image have been covered. This process is repeated iteratively until the importance of all portions of the image to the model’s decision-making process is assessed. The result is a heatmap that identifies the spectral bands relevant to the prediction and indicative of the physico-chemical oxidation process. For clarity, the heatmap is smoothed with a Gaussian filter ($\sigma = 3$ pixels), and contour lines are added to illustrate how AE increases are distributed. This technique leverages the internal feature representation of the network to study natural processes without retraining the model.

This method allows us to identify and visualise which parts of the EEMs are most critical for accurate predictions, and thus, as a proxy, to the physico-chemical process of oil oxidation, enhancing the interpretability of the DL model. This approach essentially maps the sensitivity of the model’s predictions to variations in different spectral areas of the data. This model has been inspired by existing methods, such as the backward feature elimination and *Y*-randomisation approach (see, for example,^25^), although it uses a fundamentally different approach. To understand the physico-chemical processes underlying the observations, we selectively remove bands associated with different substances and processes, such as chlorophyll (which has a fluorescence emission in the 600 nm - 750 nm range, for example) or primary and secondary oxidation products (which have a fluorescence emission in the 375 nm - 550 nm range), from the EEMs to evaluate their importance for the predictions. Assuming that the NN has developed an internal representation that models these physico-chemical processes, we can deduce which substances or processes are most influential in the oxidation of the oils.

The size of the removed region (5 $\times$ 5 pixels) corresponds to the increment in the excitation wavelengths of the original data (before reshaping). In the original EEMs (35 $\times$ 251 pixels), the sampling step for the excitation wavelength is 10 nm. 5 pixels in the reshaped matrix (160 $\times$ 160 pixels), corresponds to 11 nm (1 pixel corresponds to 2.2 nm). In the other dimension (emission wavelengths) one could also take a smaller size. However, because of the broad fluorescence features in the EEMs, this will not lead to better interpretability. Thus, we decided to choose a square shape for the region to be removed.

## Results and discussion

**Fig. 2:**
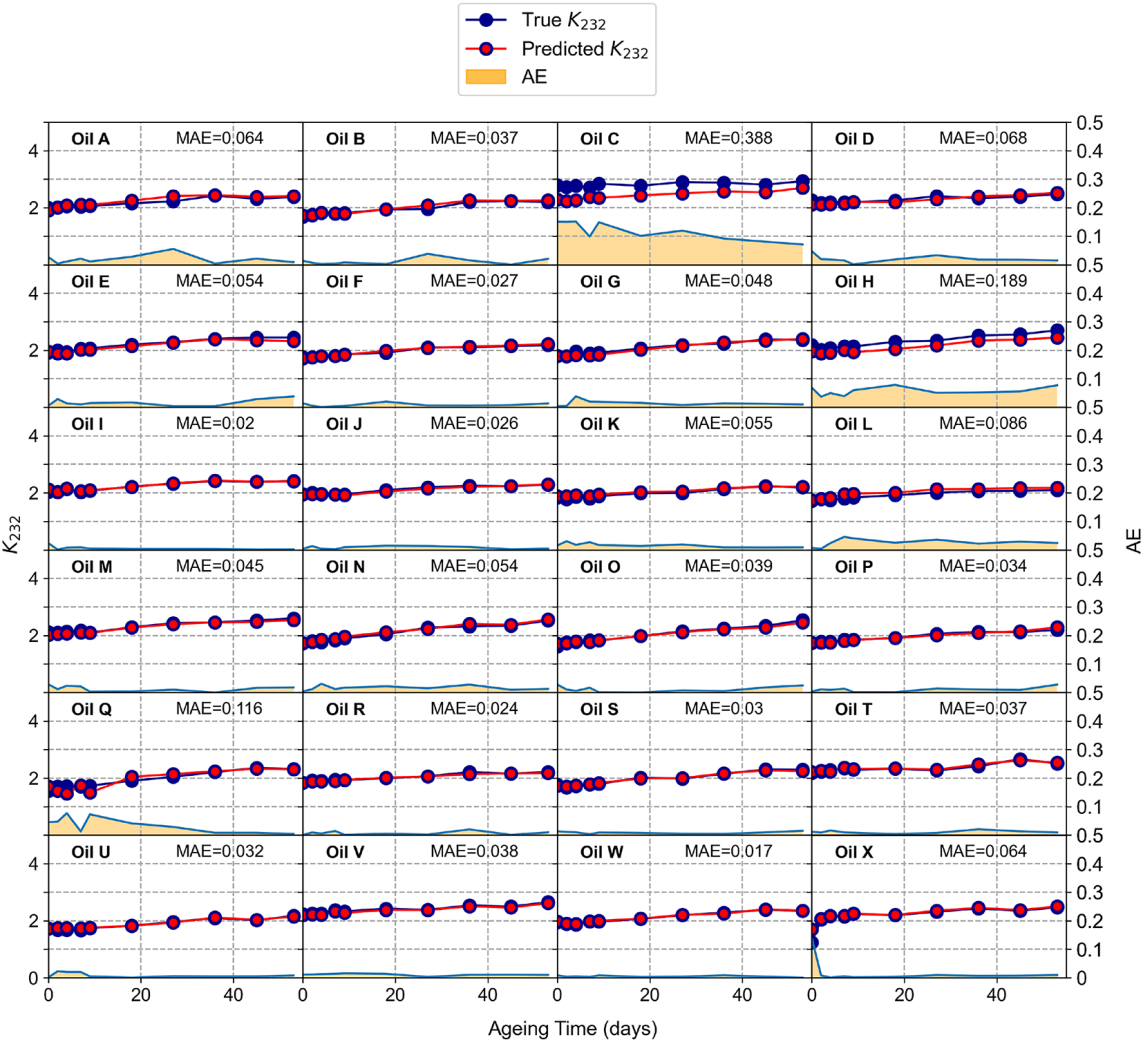
Comparison of the true (blue) and predicted (red) values of the quality indicator $K_{232}$ for all the oils at all oxidation stages (vertical scale on the left axis). The corresponding absolute error (AE) is shown as an area in yellow (vertical scale on the right axis). The Mean Absolute Error (MAE) obtained as average over all the oxidation stages is displayed in each panel for each oil.

**Fig. 3:**
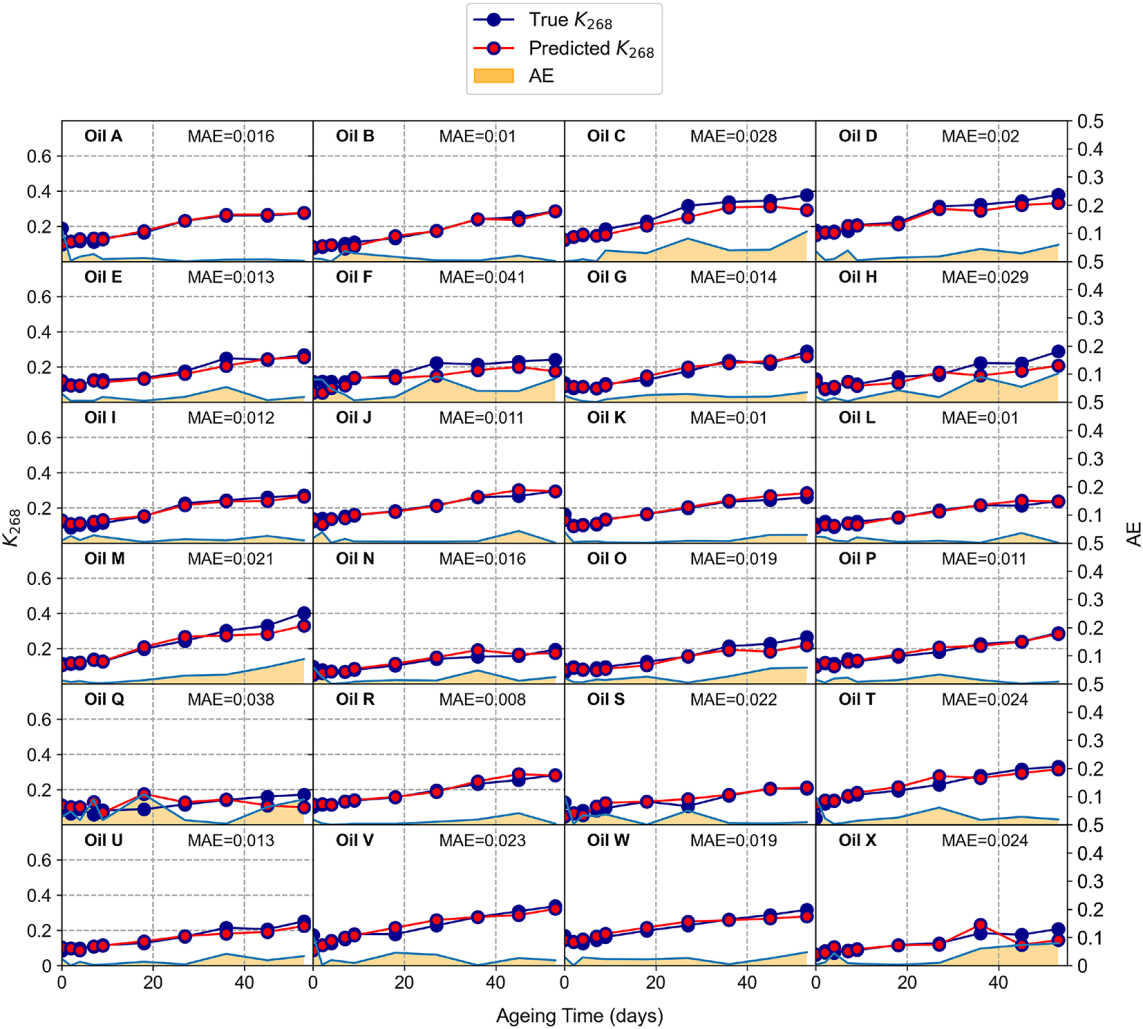
Comparison of the true (blue) and predicted (red) values of the quality indicator $K_{268}$ for all the oils at all oxidation stage (vertical scale on the left axis). The corresponding absolute error (AE) is shown as an area in yellow (vertical scale on the right axis). The Mean Absolute Error (MAE) obtained as average over all the oxidation stages is displayed in each panel for each oil.

The exceptional performance of the model in predicting the quality indicators $K_{232}$ and $K_{268}$ from fluorescence EEMs for each oil at each oxidation stage is shown in Figs. 2 and 3, where the predicted and measured values are compared directly.

**Fig. 4:**
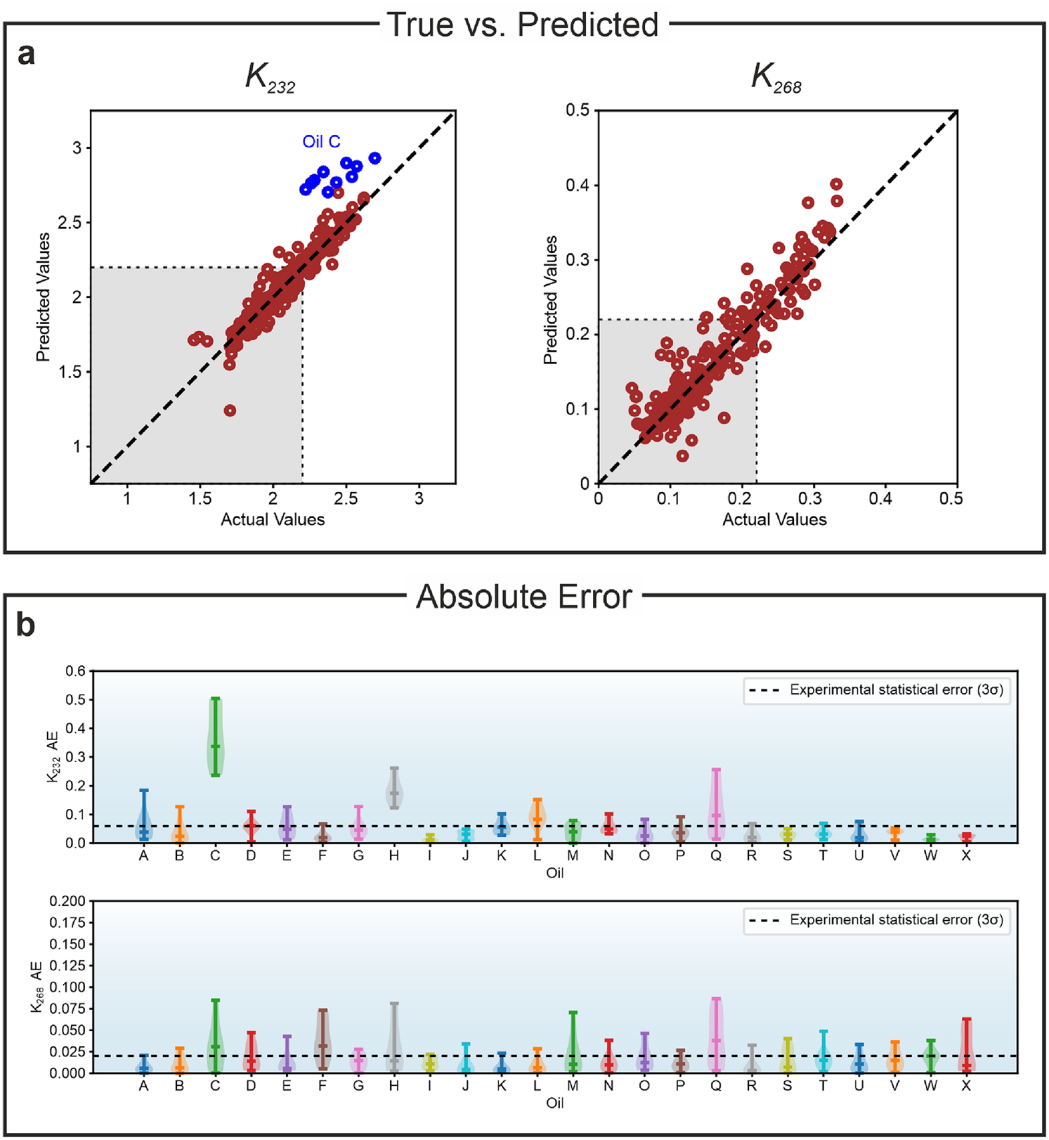
(**a**) Comparison of predicted and measured (actual) values of the quality indicators $K_{232}$ and $K_{268}$ for all oils at all oxidation stages. The gray area in each plot marks the limit set by the Food and Agriculture Organisation of the United Nations and by the European Union. Oil C is marked in blue as the $K_{232}$ value was was already above this limit at the beginning of the study and, therefore, is not well predicted by the model. (**b**) Violin plots of the AE for each oil for $K_{232}$ (above) and $K_{268}$ (below). The dashed lines indicate the 3$\sigma$ statistically estimated experimental error.

The results are summarised in Fig. 4. The exception is the prediction for oil C, marked in blue in Fig. 4a, which is, however, easily explained, as the value $K_{232}$ at the beginning of the study was already above the limit and, therefore, cannot be well predicted by the model. Fig. 4b shows the distribution of the absolute error (AE) for each oil at each oxidation stage. The total mean absolute error (MAE) is 0.066 for $K_{232}$ and 0.010 for $K_{268}$, compatible or lower than the statistically estimated experimental error, also shown in the figure.

**Fig. 5:**
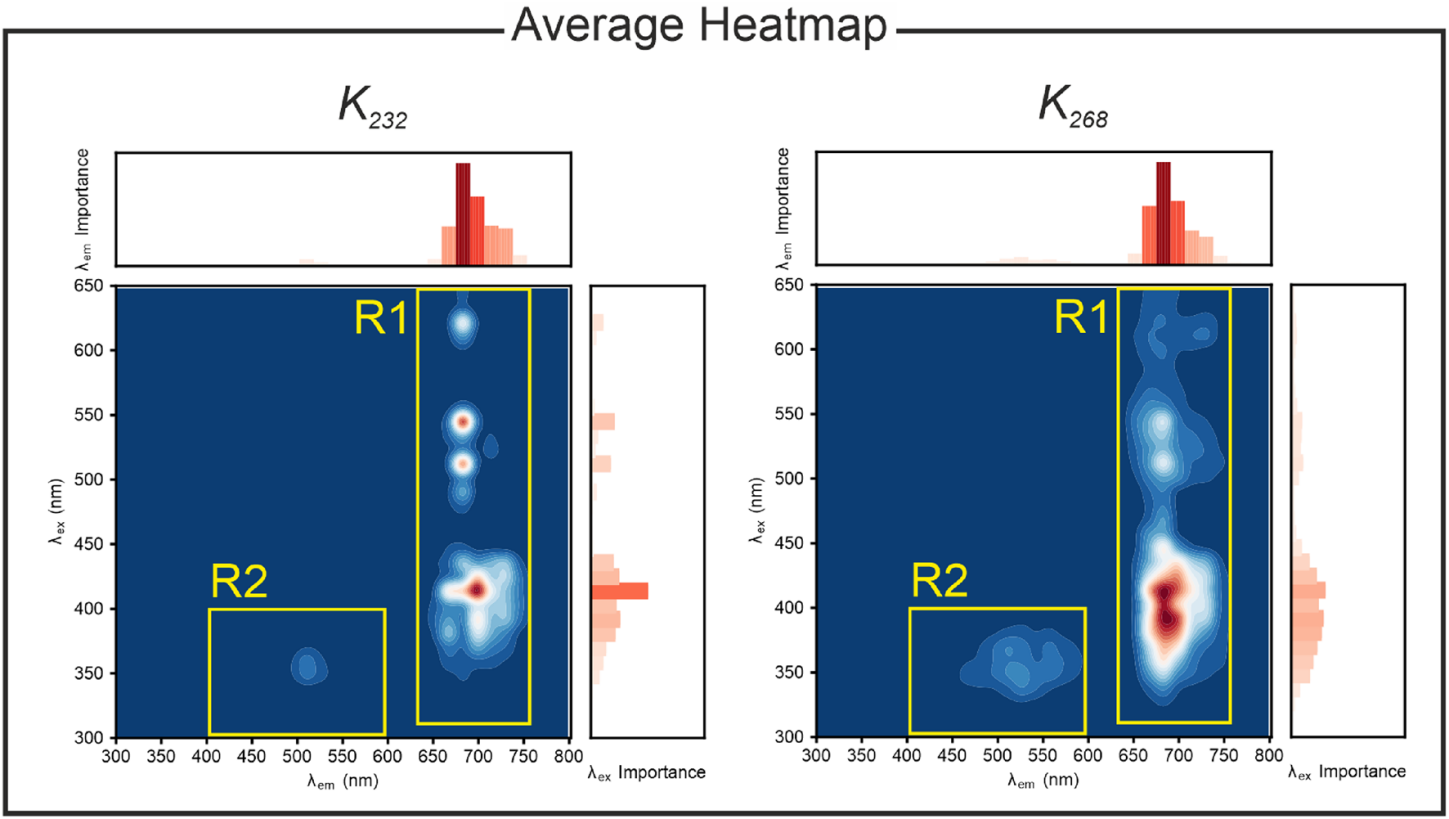
Average of the heatmaps obtained for all oils in the last oxidation stage showing the spectral band of relevance for the prediction of the $K_{232}$ and $K_{268}$. R1 marks the absorption and emission bands of chlorophylls, R2 those of oxidation products.

Once the reliability of the model has been established, the IEA allows to interpret how the model extracts knowledge from the spectral data. To determine the features that are generally relevant for all EVOO independently of the specific geographical origin or cultivar, the average of the resulting heatmap can be calculated. The result is shown in Fig. 5 for both quality indicators $K_{232}$ and $K_{268}$. The bars in the top barplot are 5 pixel wide and have a height corresponding to the sum of all the increases of the AE in the 5-pixel-wide vertical region of the heatmap that lies immediately below the bar. Each bar in the right barplot is analogously 5 pixels wide, with a height corresponding to the sum of all the increases of the AE in the 5-pixel-wide horizontal region of the heatmap that lies immediately to the left of the bar. As such, each bar measures the importance of a specific excitation (for the side barplot) and emission (for the top barplot) wavelength range for the predictions of the two parameters $K_{232}$ and $K_{268}$, respectively.

The IEA identifies two relevant spectral ranges that are associated with chlorophyll and oxidation products. The chlorophylls’ bands (absorption between 300 and 650 nm, emission between 650 and 750 nm, R1 in Fig. 5) are the most significant spectral components for the prediction of $K_{232}$. For the determination of $K_{268}$, the oxidation products (absorption between 300 and 400 nm, emission between 400 and 500 nm, R2 in Fig. 5) acquire relevance. These latter results indicate that $K_{268}$ is the most sensitive indicator of the presence of oxidation products. This is consistent with previous observations of greater changes in $K_{268}$ than in $K_{232}$ during thermal degradation^16^.

**Fig. 6:**
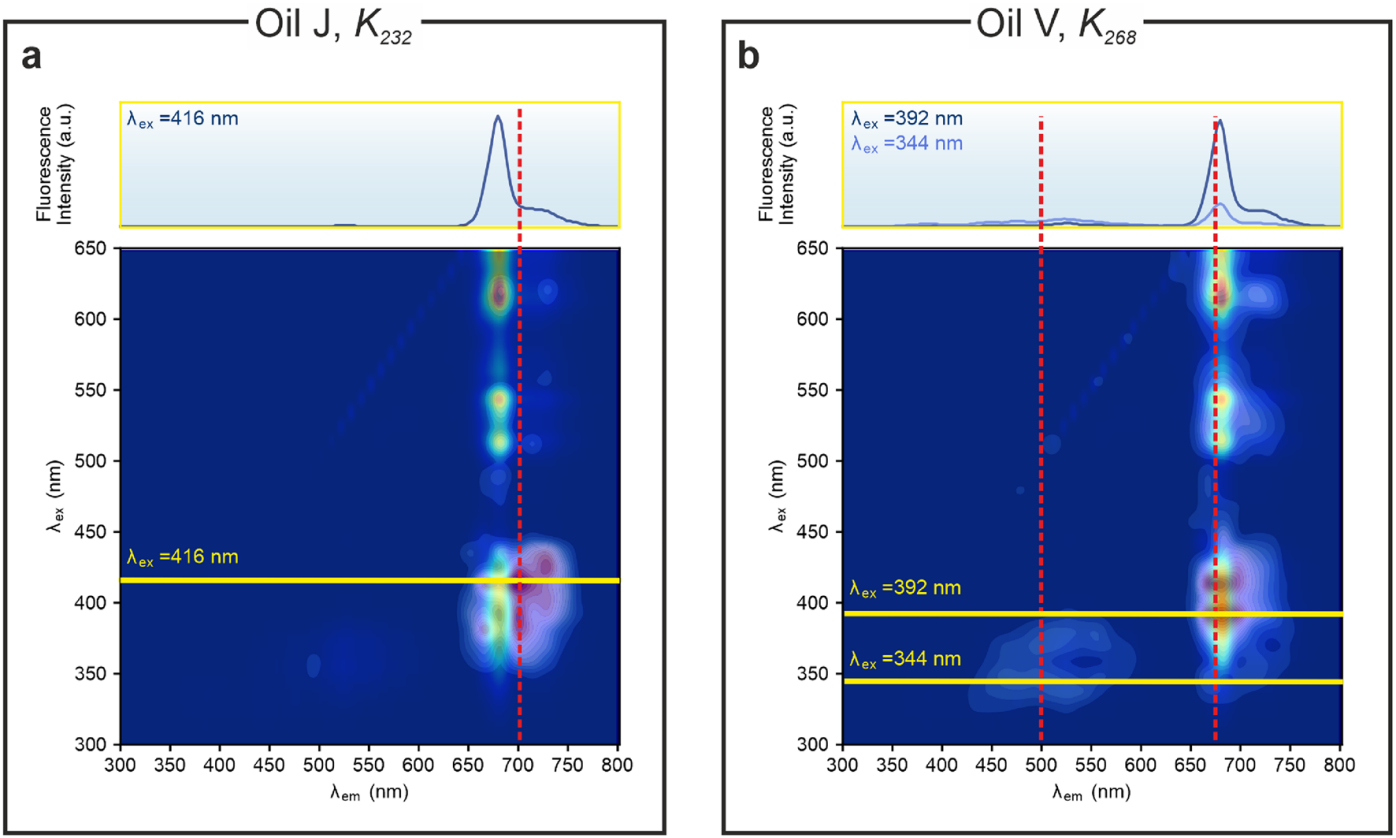
IEA approach showing the spectral bands of the fluorescence spectrum which most significantly contribute to the prediction for two selected oils in the last oxidation stage. (**a**) EEM of Oil J and region importance heatmap overimposed for quality indicator $K_{232}$. The fluorescence spectrum at $\lambda _{\text {ex}}=416$ nm is shown in the top panel. (**b**) EEM of oil V and region importance heatmap overimposed for quality indicator $K_{268}$. The fluorescence spectra at $\lambda _{\text {ex}}=344$ nm and $\lambda _{\text {ex}}=392$ nm are shown in the top panel.

The analysis of the relevant spectra features can be further pushed by overlaying the heatmap with the EEMs, as shown in Fig. 6. The IEA specifically identifies as the most relevant excitation wavelength $\lambda _{\text {em}}=416$ nm. The most relevant features of the corresponding fluorescence spectrum for the prediction of $K_{232}$ are the shoulder between the maximum chlorophyll emission at 680 nm and the broad secondary peak at 720 nm. Differently, for the prediction of $K_{268}$ the IEA identifies the most relevant excitation wavelengths $\lambda _{\text {em}}$ to be 344 nm and 392 nm. The most important feature of the corresponding fluorescence emission spectra is the maximum of the chlorophyll emission peak at 680 nm, with some contribution from oxidation products at ca. 500 nm. These findings show that the two quality indicators are affected by the presence of different chemical components.

**Fig. 7:**
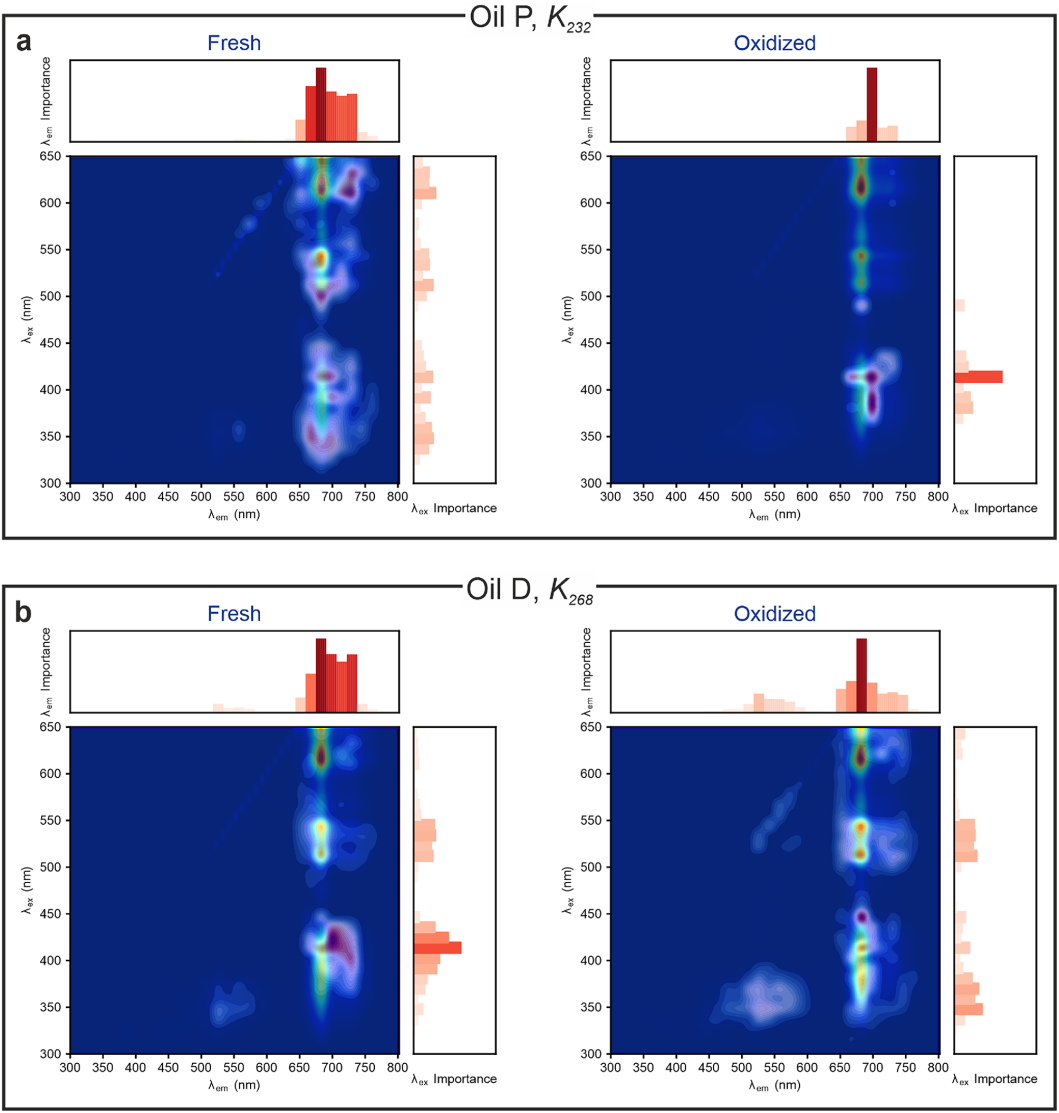
IEA approach showing the evolution during the oxidation process on two selected oils: “Fresh” marks the oil just after opening the bottle, “Oxidized” the oil at the latest oxidation stage. (**a**) Oil P EEM and region importance heatmap overimposed for the prediction of the quality indicator $K_{232}$; (**b**) Oil D and region importance heatmap overimposed for the prediction of the quality indicator $K_{232}$.

Finally, the IEA can be used to study the progression of the oxidation process by investigating the evolution of the identified spectral bands. The results of this analysis are shown in Fig. 7 for two selected oils as an example. As the oxidation progresses, the identified relevant spectral range becomes narrower, becoming visually recognisable as a heatmap that is less spread throughout the EEM (see Fig. 7a) and, if oxidation products are present, they gain increased relevance (see Fig. 7b). This means that the model becomes more precise in identifying the relevant chemical components as the features emerge more clearly.

## Conclusions

In summary, the new contributions of this work are twofold. First, we present a domain adaptation method that allows the application of DL even with a small dataset. The method is demonstrated through its application to assess the decrease in extra virgin olive oil quality during storage using fluorescence data. The model is trained on a dataset of 240 fluorescence EEM with high similarity to determine the mandatory indicators $K_{232}$ and $K_{232}$ needed for quality assessment. The results show that the model has exceptional performance and predicts the two indicators $K_{232}$ and $K_{232}$ with very high accuracy (low MAE), comparable to the experimental error. Therefore, the proposed approach allows to construct a model that is interpretable as a proxy for the physico-chemical process. This approach represents a significant innovation because it applies domain adaptation to utilise the MobileNetv2 architecture, originally designed and pretrained using conventional photographic images, for scientific applications, thus bridging the gap between classical image recognition and the specialised needs of scientific imagery analysis. Second, we introduce the IEA which allows to determine without any prior knowledge the spectral absorption and emission bands involved in the oxidation process. The spectral bands are robustly identified independently of the specifics of the olive oil and become more and more pronounced as the process progresses, as expected. This second contribution transforms DL from a black-box to an interpretative tool, thus offering a key to understanding the underlying physico-chemical process. Despite its broad applicability, this method has the limitation that the pretrained network expects two-dimensional arrays as input and as such does not work, for example, with one-dimensional spectra. Future research could expand the applicability of the method by developing an equivalent model of MobileNetV2 for one-dimensional spectra. A further research direction could include a performance comparison with models other than MobileNetV2. In general, the impact and applicability of the research presented in this work go beyond fluorescence EEMs because such an approach can be easily applied to other types of data and images.

## Data Availability

The dataset of this study is freely available for download at https://doi.org/10.17632/g6y69g8gwm.1 and is described in detail in^24^.

## References

[1] Moerner, W. & Fromm, D. P. Methods of single-molecule fluorescence spectroscopy and microscopy. *Rev. Sci. Instrum.* **74**(8), 3597–3619 (2003).

[2] Lakowicz, J. R. *Princ. Fluoresc. Spectrosc.* (Springer, 2006).

[3] Dos Santos, R. F. et al. Alzheimer’s disease diagnosis by blood plasma molecular fluorescence spectroscopy (eem). *Sci. Rep.* **12**(1), 16199 (2022). 36171258 10.1038/s41598-022-20611-y PMC9519548

[4] Sikorska, E., Khmelinskii, I., & Sikorski, M. Fluorescence spectroscopy and imaging instruments for food quality evaluation. In *Evaluation Technologies for Food Quality*, pp. 491–533. Elsevier, Philadelphia, USA (2019).

[5] Costa, F. S., Bezerra, C. C., Neto, R. M., Morais, C. L. & Lima, K. M. Identification of resistance in escherichia coli and klebsiella pneumoniae using excitation-emission matrix fluorescence spectroscopy and multivariate analysis. *Sci. Rep.* **10**(1), 12994 (2020). 32747745 10.1038/s41598-020-70033-x PMC7400627

[6] Bro, R. parafarmacia tutorial and applications. *Chemom. Intell. Lab. Syst.* **38**(2), 149–171 (1997).

[7] Murphy, K. R., Stedmon, C. A., Graeber, D. & Bro, R. Fluorescence spectroscopy and multi-way techniques. Parafac.. *Anal. Methods* **5**(23), 6557–6566 (2013).

[8] Litjens, G. et al. A survey on deep learning in medical image analysis. *Med. Image Anal.* **42**, 60–88 (2017). 28778026 10.1016/j.media.2017.07.005

[9] Michelucci, U. *Applied Deep Learning with TensorFlow 2* (APRESS Springer Nature, 2023).

[10] Meza Ramirez, C. A., Greenop, M., Ashton, L. & Rehman, I. U. Applications of machine learning in spectroscopy. *Appl. Spectrosc. Rev.* **56**(8–10), 733–763 (2021).

[11] Liu, Z. et al. A survey on applications of deep learning in microscopy image analysis. *Comput. Biol. Med.* **134**, 104523 (2021). 34091383 10.1016/j.compbiomed.2021.104523

[12] Xu, R.-Z. et al. Fast identification of fluorescent components in three-dimensional excitation-emission matrix fluorescence spectra via deep learning. *Chem. Eng. J.* **430**, 132893 (2022).

[13] Chen, A.-Q. et al. Intelligent analysis of excitation-emission matrix fluorescence fingerprint to identify and quantify adulteration in camellia oil based on machine learning. *Talanta* **251**, 123733 (2023). 35940112 10.1016/j.talanta.2022.123733

[14] Yu, H. et al. Impact of dataset diversity on accuracy and sensitivity of parallel factor analysis model of dissolved organic matter fluorescence excitation-emission matrix. *Sci. Rep.* **5**(1), 10207 (2015). 25958786 10.1038/srep10207 PMC4426691

[15] Lähnemann, D. et al. Eleven grand challenges in single-cell data science. *Genome Biol.* **21**, 1–35 (2020). 10.1186/s13059-020-1926-6 PMC7007675 32033589

[16] Venturini, F. et al. Shedding light on the ageing of extra virgin olive oil: Probing the impact of temperature with fluorescence spectroscopy and machine learning techniques. *LWT* **191**, 115679 (2024).

[17] Donat-Vargas, C. et al. Only virgin type of olive oil consumption reduces the risk of mortality. Results from a mediterranean population-based cohort. *Eur. J. Clin. Nutr.* **77**(2), 226–234 (2023). 36241725 10.1038/s41430-022-01221-3 PMC9908537

[18] Commission regulation (eec) no. 2568/91 of 11 july 1991 on the characteristics of olive oil and olive-residue oil and on the relevant methods of analysis official journal l 248, 5 september 1991. Offic. JL **248**, 1–83 (1991).

[19] Commission implementing regulation no 1348/2013 of december 17 2013. *Official Journal of the European Union* **338**, 31–67 (2013).

[20] *: Standard for olive oils and olive pomace oils. Codex Alimentarius, International Food Standards **CXS 33-1981** (1981).

[21] Ghafoorian, M., Mehrtash, A., Kapur, T., Karssemeijer, N., Marchiori, E., Pesteie, M., Guttmann, C.R., Leeuw, F.-E., Tempany, C.M., Van Ginneken, B., et al. Transfer learning for domain adaptation in mri: Application in brain lesion segmentation. In *Medical Image Computing and Computer Assisted Intervention- MICCAI 2017: 20th International Conference*, Quebec City, QC, Canada, September 11-13, 2017, Proceedings, Part III 20, pp. 516–524 (2017). Springer.

[22] Sandler, M., Howard, A., Zhu, M., Zhmoginov, A., & Chen, L.-C. Mobilenetv2: Inverted residuals and linear bottlenecks. In *Proceedings of the IEEE Conference on Computer Vision and Pattern Recognition*, pp. 4510–4520 (2018).

[23] Deng, J., Dong, W., Socher, R., Li, L.-J., Li, K., & Fei-Fei, L. Imagenet: A large-scale hierarchical image database. In *2009 IEEE Conference on Computer Vision and Pattern Recognition*, pp. 248–255 (2009). 10.1109/CVPR.2009.5206848.

[24] Venturini, F., Fluri, S. & Baumgartner, M. Dataset of fluorescence eem and uv spectroscopy data of olive oils during ageing. *Data* **8**(5), 81 (2023).

[25] Rücker, C., Rücker, G. & Meringer, M. Y-randomization-a useful tool in qsar validation, or folklore. *J. Chem. Inf. Model.* **47**, 2345–2357 (2007). 17880194 10.1021/ci700157b

